# The 2024 monkeypox outbreak in Pakistan: assessing the nation’s public health preparedness

**DOI:** 10.1097/MS9.0000000000003398

**Published:** 2025-05-20

**Authors:** Hashir Ali Awan, Muhammad Omaise Zafar, Syeda Isra Tahera, Gaffar Alemam A Manhal, Khabab Abbasher Hussien Mohamed Ahmed, Irfan Ullah

**Affiliations:** a Department of Internal Medicine, Dow Medical College, Karachi, Pakistan; b Faculty of Medicine, University of Khartoum, Khartoum, Sudan; c Department of Internal Medicine, Khyber Teaching Hospital, Peshawar, Pakistan

**Keywords:** clade Ib, epidemic, monkeypox, virus, WHO

## Abstract

The new subclade Ib of the monkeypox virus (MPXV) is spreading rapidly across Africa and has now been reported outside the continent. Historically, clade I has been associated with more severe disease than clade II, and its newly mutated variant, clade Ib is raising further concerns. Pakistan is among the first non-African countries to confirm a case of mpox in the recent outbreak, amid the World Health Organization’s sounding warning of a public health emergency of international concern (PHEIC). With an underfunded, underequipped, and overburdened healthcare system, Pakistan remains virtually unprepared for a public health emergency as a result of mpox. If sustained human-to-human transmission continues, Pakistan will face yet another public health challenge after COVID-19 and the recent devastating floods of 2022. To mitigate this crisis, a coordinated national strategy, in addition to collaboration with international organizations are imperative.

## Introduction

Mpox (previously known as monkeypox) is a zoonotic disease caused by the monkeypox virus (MPXV). It is transmitted through direct contact with infected individuals, including body fluids, lesion materials, contaminated surfaces, or respiratory droplets ^[^1^]^. The prodromal phase of the disease includes fever, headache, lymphadenopathy, chills, and myalgia, followed by the eruptive phase, characterized by a progressive rash that progresses through various stages before crusting. Severe cases can be complicated by secondary bacterial infections, pneumonia, encephalitis, and visual impairment^[^2^]^.

**Figure 1.**
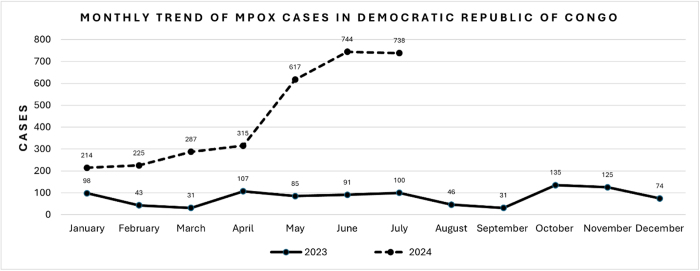
Monthly trend of mpox cases in the Democratic Republic of Congo.

On 14 August 2024, while Pakistan celebrated 77 years of independence, the World Health Organization (WHO) declared mpox a public health emergency of international concern (PHEIC)^[^3^]^. This declaration followed an alarming surge in cases reported in the Democratic Republic of Congo (DRC) and surrounding African countries. The WHO reported 500 deaths in the DRC alone in 2024, a significant escalation compared to previous outbreaks (Fig. 1) ^[^4^]^. In contrast, according to the Centers for Disease Control and Prevention (CDC) the 2022–2023 outbreak reported 207 deaths globally, raising concerns about the higher virulence and transmission of the current outbreak. However, due to underreporting and limitations in disease surveillance, the actual numbers may be higher than what was officially documented^[^5^]^.

The WHO previously declared a similar announcement in July 2022, following the rapid spread of Clade IIb, a less severe variant of MPXV. According to the CDC, nearly 100 000 global cases were reported during this epidemic, with most cases were attributed to secondary transmission among men who have sex with men. In May 2023, following a decline in cases, the PHEIC declaration was removed^[^6^]^. The current outbreak is driven by Clade Ib, a variant of Clade I, which is known to cause more severe clinical presentations and mortality^[^7^]^. In some outbreaks, clade I has been associated with a mortality rate of more than 10% among infected individuals^[^7^]^. In comparison, Clade II infections had more than 99.9% survival rate^[^1,5^]^. Thus, the emergence of this more virulent strain raised the international concerns regarding its potential transmission and healthcare systems preparedness.

While previous mpox outbreaks were largely confined to Africa, the detection of MPXV beyond the region amplified global concerns^[^7^]^. After Sweden confirmed the first European case of the current outbreak, Pakistan reported one case of mpox. Notably, Thailand has also confirmed a case of mpox on 22 August 2024^[^8^]^. Within hours of Thailand, Pakistan confirmed a second case^[^9^]^. While Sweden has determined the case is caused by MPXV of clade Ib origin, the case in Pakistan is yet to be genetically ascertained.

Global cooperation is necessary to stop MPXV epidemics through targeted vaccination, contact tracing, isolation, and early case detection. As shown in previous epidemics such as Ebola, the ring vaccination strategy, which involves immunization of close contacts to the confirmed cases, has been successful in stopping disease transmission. JYNNEOS and ACAM2000, two smallpox vaccines approved by the Food and Drug Administration (FDA), provide cross-protection against MPXV. Vaccination lowers the likelihood of infection, but it does not completely eradicate the disease. The implementation of ring vaccination strategy can be challenging due to vaccine hesitancy, and contact tracing issues, especially in underserved populations^[^10^]^. In order to stop the spread of MPXV, hygiene and protective measures are also crucial. Isolating affected individuals, adhering to hand hygiene guidelines, using masks and gloves, and sanitizing surfaces are recommended. Moreover, antiviral therapies such as tecovirimat exhibit potential; nevertheless, more investigations are required to determine their efficacy^[^11,12^]^. Additionally MPXV is now exhibiting evolving transmission dynamics, including potential cross-species and human-to-human transmission. Human-to-animal transmission has been documented, with a confirmed case of a dog in Paris contracting MPXV from its infected owners. Genetic analysis verified that the same virus strain caused the infection in both humans and the pet. This raises concerns about reverse zoonosis—MPXV adapting to new animal hosts, potentially creating reservoirs outside Africa and leading to recurring human infections^[^13^]^.

## State of Pakistan’s healthcare system

Pakistan’s healthcare system is already underfunded, overburdened, and structurally fragile, making it vulnerable to public health crises. As of 2021 according to the World Bank, Pakistan allocated 2.91% of its Gross Domestic Product (GDP) to health expenditure, one of the lowest percentages in South Asia. In contrast, Afghanistan allocated 21.83%, Iran 5.77%, China 5.38% and India 3.28% of their GDP to healthcare^[^14^]^. This disparity reveals the chronic underinvestment in Pakistan’s healthcare system, limiting its capacity to address infectious outbreaks. A recent study in Khyber Pakhtunkhwa, the province where the first mpox case was reported, revealed that the province lacked the ability to manage disasters and public health emergencies^[^15^]^. Pakistan’s historical response to health crises highlights its inability to effectively encounter outbreaks. The COVID-19 pandemic exposed critical weaknesses, including limited testing capacity, overwhelmed hospitals and delayed vaccines distribution^[^16^]^. Apart from the fragility of Pakistan’s healthcare system, the dense population, limited health literacy, and lack of adherence to hygiene protocols compound the burden of any epidemic^[^17^]^.

If mpox continues its sustained local spread within the country, it poses a significant threat to the South Asian nations. Following the 2022–2023 outbreak, researchers explicitly warned about Pakistan’s lack of preparedness for future crises^[^18^]^. It has been noted that, although Pakistan has the primary healthcare facilities needed to combat an epidemic, they are not operational due to the overcrowding in large hospitals, a lack of healthcare personnel, and challenges faced by the workforce due to cultural and linguistic limitations^[^17,18^]^.

While the National Command and Operation Centre (NCOC) of Pakistan has initiated emergency measures by tightening border checks and imposing strict infection prevention and control measures (including but not limited to hand hygiene, environmental cleaning, and disinfection), these efforts constitute only part of the solution in responding to mpox^[^19^]^. A robust vaccination drive alongside strict adherence at a national level to standard operating procedures is required to combat the threat. Vaccines for monkeypox ceased in Pakistan in the 1990s, and the recent COVID-19 vaccination drive and the associated hesitancy are key indicators that even a mass vaccination campaign will encounter roadblocks^[^18^]^. Vaccine hesitancy in Pakistan is influenced by psychosocial factors including risk perception, social stigma, and the vaccine safety rumors^[^20^]^. Addressing these challenges through targeted awareness campaigns is required beyond vaccine distribution. Thus, even if Pakistan secures mpox vaccines, adherence may remain suboptimal unless specific measures are adopted to address public concerns. Additionally, Pakistan has experienced frequent outbreaks of human immunodeficiency virus (HIV) infections, the most recent which occurred in the southern city of Larkana, where nearly a thousand cases were reported in the low-risk pediatric population^[^21^]^. All these factors combined make Pakistan a high-risk country for a mpox epidemic.

The lack of financial resources in Pakistan leads to a lack of facilities and infrastructure such as ventilators, hospital beds, and laboratory equipment, which ultimately makes it even more challenging to address infectious diseases such as mpox^[^2^]^. During the 2022–2023 outbreak of mpox, Pakistan did not have any diagnostic facilities to detect the virus; therefore, all the samples were sent abroad, which further strained the already depleted financial resources in the country^[^22^]^. Furthermore, in recent years, Pakistan has faced multiple challenges, namely COVID-19 and floods, which further drain its financial resources. With monsoons expected to ravage the country again in 2024, an efficient and timely emergency response to a potential mpox epidemic remains doubtful. The global mpox response during the previous 2022–2023 outbreak emphasized the importance of improving surveillance, government engagement, and vaccine distribution. While the WHO launched vaccine donation programs, successful community-based awareness campaigns in Germany and Peru reduced the disease transmission^[^23^]^. Pakistan could apply the lessons from the past outbreak to improve its preparedness for this emerging mpox crisis.

## Conclusion

Pakistan needs to work in close coordination with international health agencies like the WHO and Gavi. A cohesive national plan should be implemented targeting vigilant surveillance, rapid testing, contact tracing, and adequate treatment for identified cases. As per the NCOC, the country has already designated several centers for potential mpox cases but careful coordination with local healthcare networks is required for efficient implementation. Lastly, international efforts should be focused on assisting Pakistan in tackling the emerging threat.

## Data Availability

The data that support the findings of this study are available with the corresponding author upon reasonable request.

## Research Registration Unique Identifying Number (UIN)

1 Name of the registry: Not required.

2 Unique Identifying number or registration ID: Not applicable.

3 Hyperlink to your specific registration (must be publicly accessible and will be checked)
